# The attendance paradox: schools as ambivalent spaces of adolescent substance use

**DOI:** 10.3389/fpubh.2026.1920425

**Published:** 2026-09-18

**Authors:** A. Abinaya, V. Vijayalakshmi

**Affiliations:** School of Social Sciences and Languages, VIT University Chennai, Chennai, India

**Keywords:** adolescents, education, Indian government schools, substance use, Tamil Nadu, teachers, thematic analysis

## Abstract

**Background:**

Adolescent substance use in India has become a significant public health challenge. It is increasingly characterized by a shift toward easily concealed smokeless tobacco pouches (“Cool Lip”) and inhalants, which fall outside awareness-based school prevention frameworks. School absenteeism has been identified in the global literature as the key behavioral indicator of adolescent substance use, based on the assumption of a relatively stable home environment. This assumption may not hold in low-income urban government schools in India, where the Right of Children to Free and Compulsory Education (RTE) Act, 2009, legally requires that every child enrolled be retained. This study investigates this question from the perspective of government school teachers.

**Methodology:**

Semi-structured in-depth interviews were conducted with twenty-one government school teachers, headmasters, and physical education instructors across 16 schools in Chennai district, Tamil Nadu, India. Data were analyzed using reflexive thematic analysis in combination with a six-step conceptual model development process, with theoretical frameworks introduced only after the inductive generation of themes.

**Results:**

Five themes were identified. In contrast to the global pattern, substance-using students were consistently described as regular attendees—the Attendance Paradox. The substance landscape has shifted toward easy-to-hide substances, reshaped by post-pandemic mask use and digital peer coordination through mobile phones acquired for online learning. Teachers described a porous school perimeter where making complaints was perceived as personally risky, thereby sustaining institutional silence. Family conditions were an expression of economic precarity, not parental neglect. Teachers prioritized relational inclusion over exclusionary discipline because they perceived that excluding students could increase their vulnerability to becoming “social elements” who might subsequently influence younger peers.

**Discussion:**

The findings are synthesized into the Institutional Ambivalence Model (IAM), an empirically grounded conceptual model that explains the Attendance Paradox by showing how urban government schools simultaneously function as spaces of care and exposure. The model thereby extends sociological ambivalence from individual social roles to institutions. Policy implications support reintegration-first approaches, attention to post-pandemic digital coordination, and safer perimeter-reporting channels over attendance monitoring or exclusionary discipline.

## 1 Introduction

Adolescent substance use is a significant global public health concern that affects individual development and disrupts the social networks, including families, neighborhoods, and schools—that structure young people’s everyday lives. Over the past two decades, educational institutions have come to occupy a uniquely complex position within this shifting terrain. Macro-level evaluations of the COVID-19 pandemic indicate that it did not produce a uniform global increase in substance use but instead altered patterns of consumption, increasing risks and producing localized spikes among already marginalized young people (1).

School absenteeism is a recognized behavioral indicator of adolescent substance use in the global educational research literature (2, 3). International longitudinal data suggest that absenteeism and substance use constitute a mutually reinforcing feedback loop that intensifies during adolescence (3). This foundational body of research is predicated on a largely unexamined assumption about the spatial and social conditions of the home environment represent a relatively stable, well-supervised, and secure environment against which school attendance can be effectively evaluated (2, 3). When educational institutions employ exclusionary disciplinary practices such as suspension and expulsion, they may inadvertently generate adverse outcomes by displacing at-risk students into unsupervised community environments (4). A significant conceptual disparity exists between this international literature and the institutional constraints of government schools in urban India. A significant proportion of children in state-funded schools in India come from low-income families, single-parent households, and marginalized communities with high levels of economic instability (5). The Right of Children to Free and Compulsory Education (RTE) Act, 2009, requires schools to refrain from exclusionary disciplinary practices and retain all enrolled children (6). In such contexts, where neighborhood environments and extended parental work hours may weaken home supervision, the fundamental premise of western absenteeism models may not be relevant. The assumed association between substance use and absenteeism may not hold if the school environment is more structured, predictable, and physically safe than the home or community. This study addresses this empirical and theoretical gap by investigating how government school teachers in Chennai, Tamil Nadu, navigate substance-related risks within specific structural restrictions. Accordingly, this study was guided by the following three research questions:

*RQ1*: What are the specific substances, physical locations, and behavioral patterns of use that teachers observe among their students?

*RQ2*: How do teachers perceive the school’s role in relation to students’ home and neighborhood environments, and what institutional and relational practices do they use to support students exposed to substance-related risks?

*RQ3*: How do patterns of school attendance and the association between substance use and absenteeism in this urban Indian setting compare with those reported in the international literature, and what structural forces explain the differences?

## 2 Literature review

### 2.1 The changing landscape of substance use among Indian adolescents

The national-level picture of adolescent substance exposure in India is primarily drawn from survey data (7). Shifting focus to localized institutional environments, Batra et al. (8) conducted school-based surveys to investigate patterns and correlates of substance use among school-going students throughout India, revealing a strong association between peer substance use and subsequent tobacco and cannabis use. The study also reported a low perception of harm regarding volatile inhalants among school-going adolescents. Biswal et al. (9) conducted semi-structured interviews with adolescents in drug-treatment settings and healthcare providers across three district regional sites in India, identifying peer influence, unstable domestic environments, and systemic socioeconomic instability as primary drivers of substance progression. However, since their sample was limited to treatment-seeking adolescents, the school as an institutional setting remains unexplored.

Smokeless tobacco poses a uniquely acute and localized challenge for educational institutions (29). A survey of children in residential schools in central India found that the primary factors for smokeless tobacco use were low retail cost, immediate proximity of vendor outlets to school perimeters, and active peer and parental modeling (10). Dsouza et al. (11) assessed media reporting on gutka ban enforcement, highlighting persistent gaps between legislative prohibition and the implementation of smokeless tobacco control, while drawing attention to the complexity of regional and local supply chains that undermine enforcement. Volatile inhalants from common household products and school supplies are another readily available, inexpensive, and unregulated form of substance use among school-aged adolescents (8, 12) in addition to the risks of commercial tobacco. The scale of this enforcement challenge in Tamil Nadu has received judicial attention. In Aunestraja (13) the Madras High Court (Madurai Bench) described an “extreme emergency” arising from increasing tobacco use among school children, specifically identifying “Cool Lip” as a product contributing to this trend. The Court observed that at least ten bail applications relating to tobacco seizures in and around schools were being heard each day and highlighted two structural concerns: the movement of tobacco products from neighboring states where such products remain available and the inadequacy of existing enforcement mechanisms despite the regulatory provisions under Cigarettes and Other Tobacco Products Act (COTPA), 2003. These judicial observations independently corroborate the structural conditions described by participants, namely the continued availability of tobacco products around schools and the limitations of existing enforcement mechanisms.

### 2.2 School-based prevention: design and evaluation

The Indian school-based response to drugs, through the Nasha Mukt Bharat Abhiyaan campaign and its interdepartmental guidelines, is mostly awareness-based and uses morning assemblies, educational posters, and organized anti-drug discussions to decrease consumption by raising students’ awareness of health risks (14). Pattojoshi and Tikka (15) in their critical review of school-based prevention in India argue that this model has underutilized the international evidence base relevant to comprehensive life-skills training and multi-component prevention frameworks that focus on the social and environmental conditions of use rather than on knowledge acquisition. The Indian prevention literature has not generally employed an intervention design process that actively engages government school teachers as primary informants. Their direct and contextual observations of substance use can inform the prevention model rather than limit educators to the role of passive implementers of a centrally devised awareness curriculum.

The gap between policy and practice at the school boundary also reflects a fundamental tension between two legislative regimes. The sale of tobacco products to individuals under 18 years of age and within 100 yards of any educational institution is prohibited under Sections 6(a) and 6(b) of the Cigarettes and Other Tobacco Products Act (COTPA), 2003. Schools are required to report violations to the local authorities under the Ministry of Health and Family Welfare’s (16) Tobacco-free Educational Institutions (ToFEI) regulations. The RTE Act, 2009, mandates schools to retain all children enrolled and limit exclusionary forms of school discipline. Schools simultaneously function as a reporting body under COTPA and as a retaining institution under the RTE Act, with no clear guidelines on how to deal with situations where a student is involved in tobacco-related issues and a beneficiary of the non-exclusion obligation. The current study’s findings on the “porous perimeter” show, from a grassroots perspective, the underlying structural tension created by these dual legislative requirements.

### 2.3 The institutional nature of government schools under structural marginality

The most relevant institutional analysis is provided by Balagopalan (5, 17) which details how in the post-liberalization era government schools in India have increasingly served student populations largely from low-income, marginalized backgrounds as affluent demographics have moved to private education. This structural shift is reflected in the legislative framework of RTE Act, 2009 which initially specified that no child is to be detained or expelled till the completion of class 8, irrespective of the students’ academic achievement. However, Balagopalan’s (5, 17) story is conceptual and historical, and does not involve any fieldwork with classroom educators, and does not explicitly engage with the issue of adolescent substance use. The present study extends this institutional analysis through an empirical investigation of teachers’ experiences and responses to adolescent substance use.

### 2.4 Substance use and school attendance: an international perspective

A large international literature has repeatedly found a relationship between school disengagement and teenage substance use (2, 3). Henry and Thornberry (3) demonstrated, using long-term longitudinal data from a United States cohort, that rates of truancy and substance use patterns increase together during adolescence with each behavior reinforcing the other. An integrative analysis of multiple independent datasets regarding school absenteeism was conducted by Gakh et al. (2) and found support for an association between active substance use and reduced classroom attendance across a diverse array of educational contexts. Exclusionary disciplinary practices such as suspension and expulsion have been documented in North American schools and lead to the migration of at-risk students into the surrounding community (4). Although these studies were methodologically rigorous, most were conducted in North American contexts where they assumed a relatively stable and secure home environment (2, 4). The broader literature offers little empirical evidence as to whether the absenteeism-substance use dynamics identified by Henry and Thornberry (3) and Gakh et al. (2) hold in a fundamentally different institutional context—that of a low-income urban Indian neighborhood served by a state government school with a strict inclusion mandate—where the relative stability of the home environment compared to the school environment may be completely inverted.

### 2.5 Synthesis and research gap

These four bodies of literature reveal three intersecting empirical and theoretical gaps. First, while the adolescent substance use landscape within Indian educational institutions has been quantitatively documented, it has been examined primarily from epidemiological perspectives rather than through the lens of frontline institutional actors (7, 8, 29). There is consequently a lack of data reflecting the perspective of classroom teachers, who are present during the hours of greatest student exposure and who are structurally positioned to observe this landscape firsthand.

Second, although the prevention design literature heavily critiques traditional awareness-raising campaigns, alternative multi-component proposals have not yet incorporated the situated accounts of Indian educators directly into the intervention-building process (15).

Third, the sociological literature defining the structurally marginalized position of the Indian government school has not yet been brought into theoretical conversation with international absenteeism frameworks (2, 3, 5, 17). As a result, the critical question of whether dominant Western findings regarding truancy generalize to highly vulnerable public school contexts in India remains entirely unanswered. The present study addresses these gaps by examining teachers’ accounts of adolescent substance use within urban government schools in Chennai and by developing an empirically grounded conceptual model explaining how schools simultaneously function as spaces of care and exposure.

## 3 Theoretical framework

Three established theoretical frameworks converge to structure the analysis in this study. They are introduced here and applied analytically in the discussion. The frameworks are positioned against the macro-structural backdrop of post-liberalization Indian state schooling (5, 17) and the legal-institutional framing of the RTE Act, 2009 (6). This socio-political backdrop is not a fourth analytical lens; it is the foundational structural condition that makes the institutional configuration described by all three frameworks possible (see Figure 1).

**Figure 1:**
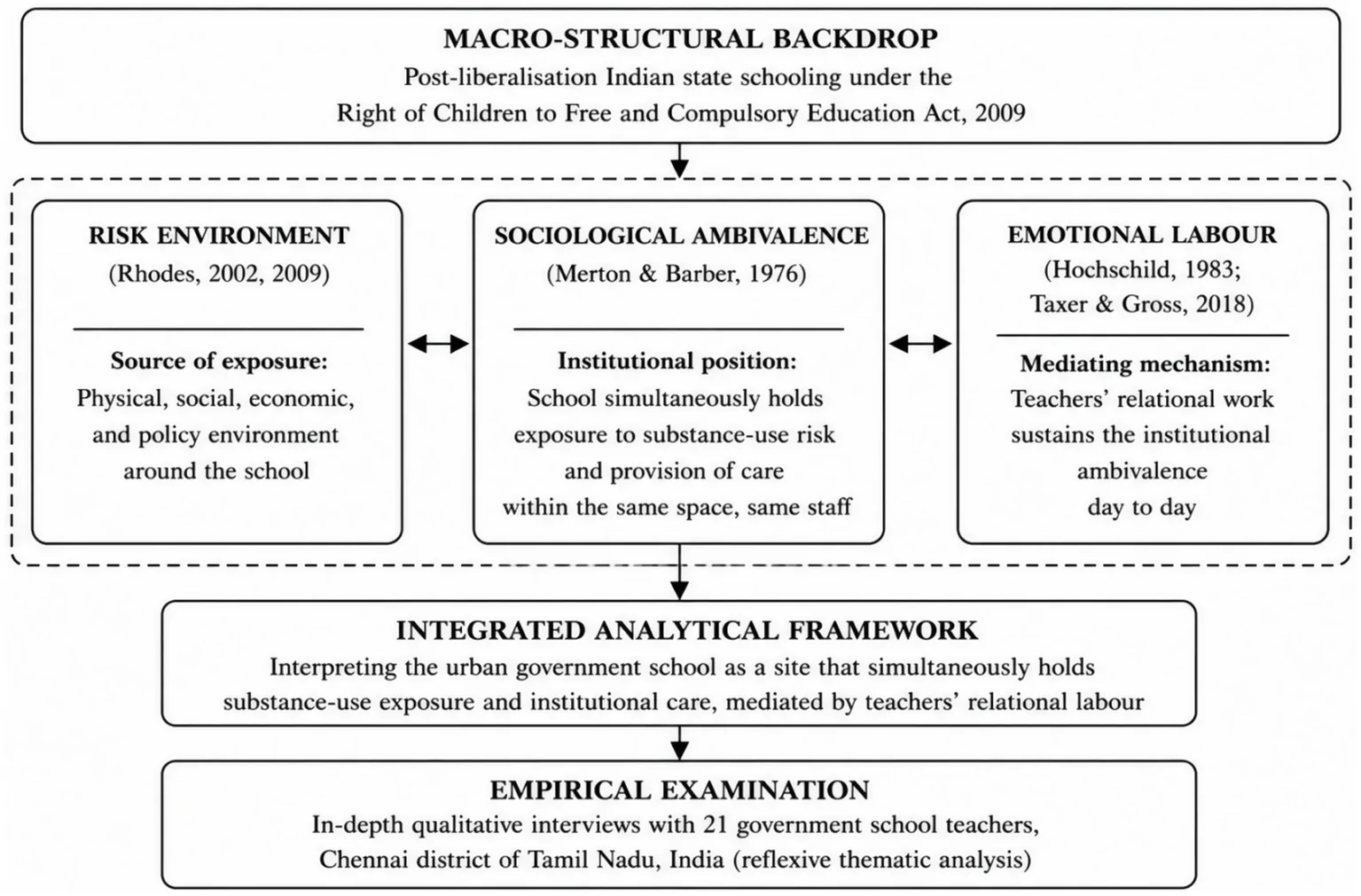
Integrated theoretical framework.

### 3.1 Risk environment

Rhodes (18, 19) developed the Risk Environment framework in critical drug sociology to shift the analytical emphasis from the individual user to the physical, social, economic, and policy context in which substance use occurs. The framework identifies four interacting dimensions: the physical environment (e.g., architecture, layout, sightlines), the social environment (e.g., peer composition, supervisory density), the economic environment (e.g., availability and cost of substances), and the policy environment (e.g., regulation and enforcement). This study employs the framework as an analytical lens for understanding where the broader adolescent substance-use environment intersects with the school environment and for explaining why formal enforcement mechanisms are perceived as costly rather than effective.

### 3.2 Sociological ambivalence

Merton and Barber (20) introduced the term sociological ambivalence to describe situations in which a single social role is structurally obliged to satisfy two legitimate but conflicting normative expectations simultaneously. The paradigm case is the physician, who must remain simultaneously detached (for clinical objectivity) and engaged (for the therapeutic relationship); this tension is an inherent property of the role’s structure, not a personal failing of the role-occupant. The present study extends this concept from social roles to institutions, proposing that the urban government school as an institution is structurally obliged to hold both exposure to substance-use risk and the provision of care within the same space and with the same staff. The simultaneous coexistence of both functions is treated as the defining institutional position, rather than as an indicator of dysfunction.

### 3.3 Emotional labor

Emotional labor is a concept developed by Hochschild (21) to refer to the managed, and often unrecognized, affective work that institutional actors undertake as part of their professional roles. Hochschild (21) distinguishes between surface acting (displaying an institutional emotion that is not genuinely felt) and deep acting (actively cultivating the required feeling within oneself). Taxer and Gross (22) translated this concept to educational settings, demonstrating the extensive emotional labor involved in managing classroom emotions, responding empathetically to individual student crises, and de-escalating behavioral confrontations. They identified that teachers engage in both surface acting—displaying institutional emotions not genuinely felt and deep acting, actively cultivating required feelings within themselves. In this study, surface acting is visible in teachers’ formal, measured responses when students are identified; deep acting is visible in the sustained relational investment, described across Theme 4 and 5 through which teachers maintain genuine commitment to students they know to be at risk. The relational practices documented in this study align with the emerging domain of relational pedagogy, defined as the deliberate fostering of teacher-student relationships as a basis for learning and wellbeing (23). In the Indian context, Parvez and Madan (24) demonstrate how close teacher-student relations operating across intersections of class, caste, and disability function as the primary mechanism through which inclusive education is made practically possible in low-resource settings. The present study extends this understanding by documenting these relational behaviors specifically in relation to substance use risk, a context in which their protective role has not previously been described in Indian educational literature.

### 3.4 The integrated theoretical framework

Figure 1 presents the three frameworks as converging analytical inputs situated against the macro-structural backdrop described above. Rhodes’ (18, 19) Risk Environment framework explains how the school’s physical, social, economic, and policy surroundings constitute Function A (exposure). Merton and Barber’s (20) sociological ambivalence explains how the school simultaneously holds Function A and Function B (care) without structural resolution. Hochschild’s (21) emotional labor explains the mechanism, teachers’ relational work—through which both functions are sustained in practice. Together, these frameworks provide the analytical foundation for interpreting the Institutional Ambivalence Model (IAM), which is developed inductively from the empirical findings in Section 6.

## 4 Materials and methods

### 4.1 Research design

The study employed a qualitative exploratory-descriptive design. A qualitative approach was chosen because the research questions concern teachers’ situated observations, accumulated over years of classroom experience, best accessed through open-ended, conversational interviewing rather than through fixed-response instruments. Data were analyzed using reflexive thematic analysis (25) in conjunction with the six-step process for developing a conceptual model from qualitative findings, described by Naeem et al. (26).

Although reflexive thematic analysis and the Naeem et al. (26) process differ in orientation, the former being explicitly committed to researcher subjectivity and the inductive meaning generation, the latter providing a structural pathway from themes to conceptual model, they were applied sequentially rather than simultaneously. Themes were generated through reflexive thematic analysis first; the six-step process was then used to organize and formalize the development of the conceptual model at the later stage of analysis. Importantly, the theoretical frameworks outlined in Section 3 were introduced only at the conceptualization stage, after themes had been generated inductively from the data, to avoid theory-led coding at an earlier stage.

### 4.2 Sample

The fieldwork was conducted in 16 government schools in Chennai, Tamil Nadu, catering to students from low-income households, including single-parent families and households in slum or resettlement areas. Participants were teachers, physical training instructors, headmasters, and Block Resource Teacher Educator (BRTE). The inclusion criteria were (i) currently employed in a government school within the study district; (ii) a minimum of 5 years of teaching or resource experience, to ensure that participants had observed patterns across time; and (iii) willingness to discuss direct observations of student behavior relevant to substance use, in confidence. Participants were recruited through purposive sampling, supplemented by snowball sampling, through which initial participants identified colleagues in specialist roles such as Physical Training instructors and the BRTE that purposive sampling alone would not reliably reach.

The final sample consisted of 21 participants. Data sufficiency was assessed through the principle of information power (27): given the study’s narrow and specific aim, the homogeneous institutional setting, the use of individual in-depth interviews, and the strong theoretical framework guiding analysis, 21 participants were considered sufficient to generate a conceptual model with analytical depth. However, as participants were recruited through purposive and snowball sampling, the findings may reflect the experiences of teachers who were more willing to discuss this sensitive topic. Years of experience ranged from 9 to 35 years (mean = 20.8 years). Participants worked in girls’, boys’, and co-educational schools. Participant characteristics are summarized in Tables 1 and 2.

**Table 1:** Participant characteristics (*N* = 21).

Code	Role	Subject specialization	Years of experience	Grades taught	School type	Gender
P1	Headmaster	Mathematics	26	9-10	Co-ed	Male
P2	Class teacher	Science	25	9-10	Girls	Female
P3	Class teacher	Biology	21	11-12	Girls	Female
P4	Class teacher	Physics	9	11-12	Girls	Female
P5	Class teacher	Geography	19	11-12	Co-ed	Female
P6	BRTE	Tamil	16	—	—	Male
P7	Class teacher	History and Geography	35	11-12	Boys	Male
P8	Class teacher	Botany	23	11-12	Girls	Female
P9	Class teacher	Mathematics	13	9-10	Boys	Male
P10	Class teacher	English	22	6–10	Co-ed	Female
P11	PT instructor	Physical Education	21	—	Co-ed	Male
P12	Class teacher	Botany	22	11-12	Girls	Female
P13	Headmaster	Chemistry	25	11-12	Boys	Female
P14	Headmaster	Zoology	21	11-12	Co-ed	Male
P15	Class teacher	Mathematics	24	11-12	Boys	Male
P16	Class teacher	Science	17	9-10	Co-ed	Female
P17	PT instructor	Physical Education	19	—	Co-ed	Male
P18	Class teacher	Social Science	14	9-10	Girls	Female
P19	Class teacher	Physics	23	11-12	Co-ed	Female
P20	Class teacher	Tamil	19	11-12	Co-ed	Male
P21	Class teacher	Zoology	23	11-12	Girls	Female

BRTE, block resource teacher educator; PT instructor, physical training instructor. Headmasters’ listed subjects reflect their teaching background prior to and/or alongside administrative duties. Grades taught exclude cross-school resource roles (P6, P11, and P17), for whom grade range is not applicable.

**Table 2:** Consolidated summary of participant characteristics.

Characteristic	Category	*n* (%)
Role	Class teacher	15 (71.4%)
	Headmaster	3 (14.3%)
	Physical training instructor	2 (9.5%)
	Block resource teacher educator	1 (4.8%)
Gender	Female	12 (57.1%)
	Male	9 (42.9%)
School type^a^	Co-educational	9 (45.0%)
	Girls’	7 (35.0%)
	Boys’	4 (20.0%)
Subject area^b^	Science (Biology/Botany/Zoology)	7 (33.3%)
	Mathematics	3 (14.3%)
	Physical sciences (Physics/Chemistry)	3 (14.3%)
	Social sciences (History/Geography/Social Science)	3 (14.3%)
	Languages (English/Tamil)	3 (14.3%)
	Physical education	2 (9.5%)
Years of experience	Range	9–35 years
	Mean	20.8 years
Grades taught^c^	Range	Grades 6–12 (predominantly 9–12)

a School type excludes the BRTE (P6), for whom school type is not applicable (*n* = 20).

b Subject area reflects participants’ teaching specialization prior to and/or alongside their current role.

c Grades taught exclude cross-school resource roles (P6, P11, and P17), for whom grade range is not applicable.

### 4.3 Research instrument

A semi-structured interview schedule was developed for this study, constructed directly from the research questions and the literature reviewed in section 2, and organized around five domains: (1) the substance landscape; (2) the social and retail environment surrounding the school; (3) family circumstances; (4) teachers’ relational and disciplinary responses; and (5) views on existing policy and support arrangements. Questions were open-ended with optional probes, and the schedule was applied flexibly; the order and depth of follow-up were determined by what each participant raised, in line with the conversational, iterative interviewing approach advocated in reflexive thematic analysis (25). The schedule functioned as a living document that evolved through engagement with the data: interview data were reviewed to confirm that questions were generating data relevant to the research questions, and slight modifications in wording were made.

### 4.4 Data collection

Following ethical approval and individual informed consent, all interviews were conducted face-to-face at the participant’s school, during free periods or after school hours, and lasted between 45 and 90 min. Most interviews were conducted in Tamil, with code-switching into English for administrative and academic terms, reflecting the bilingual register in which these issues are routinely discussed in this setting. All quotations used in the analysis were translated into English by the first author, a first-language Tamil speaker, and reviewed by a bilingual English professor proficient in both Tamil and English to ensure conceptual accuracy while preserving the meaning and register of the original.

### 4.5 Ethical considerations

The study was reviewed and approved by the institutional ethics committee at Vellore Institute of Technology, Chennai, with reference number Ref. No. VIT/IECH/CC/2026/144. All participants were informed of the purpose of the study. Participation was voluntary; participants were informed of their right to withdraw at any time without consequence. Confidentiality was maintained throughout: no personally identifiable information relating to students, families or third parties is reported. Audio recordings and transcripts are stored on encrypted, password-protected devices accessible only to the researcher. No students were interviewed.

### 4.6 Data analyses

All twenty-one transcripts were analyzed manually by the first author. Analysis was conducted in four passes using reflexive thematic analysis (25). The first pass was descriptive and inductive, remaining close to participants’ own language. The second pass clustered initial codes into preliminary themes. The third pass tested themes against the full corpus, actively seeking disconfirming cases and minority perspectives. The fourth pass moved from themes to conceptualization, following the six-step process of Naeem et al. (26): selection of representative quotations; identification of keywords within quotations; coding; theme development; conceptualization; and development of a conceptual model.

To enhance the trustworthiness of the analysis, several strategies were employed. First, observational notes recorded immediately after each interview, coding notes, and interview audio recordings were revisited throughout the analytical process to support the interpretation of participants’ accounts. Second, emerging codes, themes, and interpretations were discussed iteratively with the research supervisor to refine thematic development and critically examine emerging interpretations. This supervisory review helped to identify and challenge potential assumptions embedded in the coding process. Third, the first author maintained a reflexive journal documenting how personal and professional experiences may have shaped interpretive decisions, particularly regarding the framing of teachers’ responses as either “relational” or “disciplinary”. Member checking was not undertaken because of the sensitive nature of the topic and the need to protect participants’ confidentiality.

### 4.7 Analytical process figure

The conceptual analysis is shown in Figure 2:

**Figure 2:**
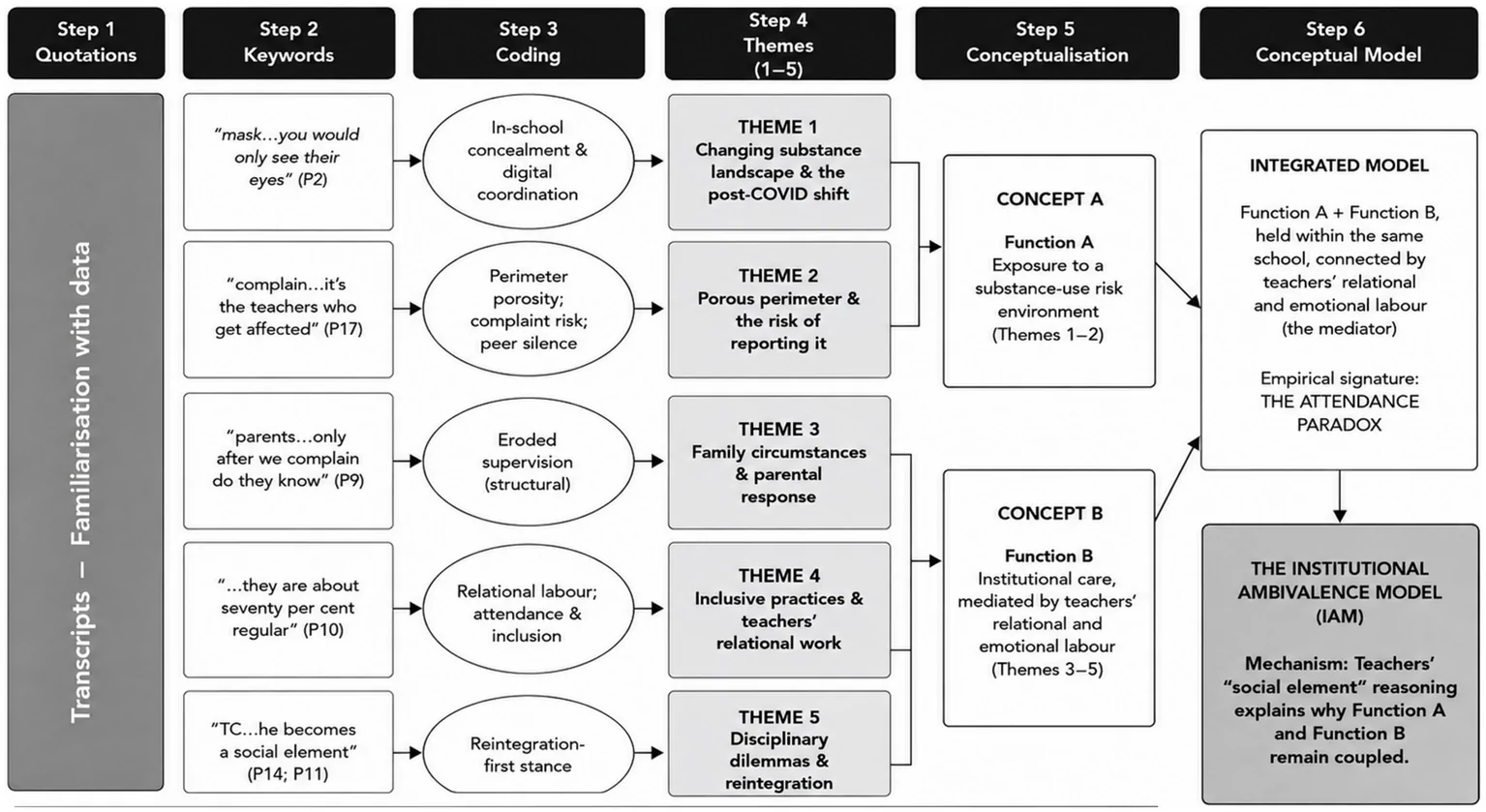
Analytical process of the conceptual model.

## 5 Results

Through the analytic process described in Section 4.7, five themes were generated. They are presented here in the sequence that best constructs the overall conceptual argument, moving from the substance landscape (Theme 1) through the institutional perimeter (Theme 2) and the home environment (Theme 3), to teachers’ relational responses (Theme 4 and 5). The participant codes (P1–P21) are retained for traceability.

### 5.1 Theme 1: a changing substance landscape and the post-COVID shift

Participants across the sample described a substance landscape that has shifted considerably over the course of their careers, moving away from older forms of alcohol and tobacco use toward newer, more easily concealed substances. Long-serving teachers described a clear historical progression: from an earlier period in which pan parag and similar products were seen only occasionally, to a present period dominated by Cool Lip, a small, pillow-shaped pouches of smokeless tobacco as well as cigarettes, beedis, and inhaling solutions such as correction fluid and cough syrup, and in a small number of more recent accounts, cannabis-based preparations. What makes Cool Lip different from previous substances is its concealability: participants noted that it was small enough to be hidden in a shirt pocket, inside a pen casing, under inner clothing, or behind a desk fitting, making routine bag and uniform checks an increasingly unreliable proxy for actual use.

A second, more striking finding was concerned with the COVID-19 pandemic, not just as a period during which usage merely increased or decreased, but as a period that changed how usage was concealed, and how students coordinated around it. One teacher described how the mask, once mandated in classrooms, gave students a new way to cover up in class:

“In the days when you had to wear a mask in class, they’d put it in, and you’d only see their eyes in that dazed state, leaning on the desk. In some classes you would see some students just go flat.” (P2).

Another participant explained a second, less obvious consequence of the pandemic: the shift to online classes meant that for the first time, almost every student had a personal mobile phone and internet access, paid for by parents specifically for online learning.

“Before, not every student had a mobile in hand. But the government said online classes were needed, so parents, even if they couldn’t really afford it, bought mobiles and gave them to their children. So now everyone has a mobile, everyone has internet, and that has given all the students a way to connect with each other. If one student knew where to get something, he could call another student immediately and inform. That kind of contact became very easy at that time, and it continued even after school reopened.” (P2).

In summary, these two observations reframe the pandemic not as a shock to substance-use rates, but as an infrastructural change. It altered the physical conditions of concealment inside the classroom (the mask) and, separately, the social conditions of coordination outside of it (near-universal student access to mobile phones bought for an entirely different, education-related purpose, and never taken away afterwards). Both changes are described as still in effect at the time of the interview, as enduring features of the post-pandemic school, not a temporary disruption.

### 5.2 Theme 2: a porous perimeter and a complaint process that teachers are reluctant to use

The second theme shifts focus from the school’s interior to its immediate surroundings. Several schools were situated close to liquor outlets and small grocery and stationery shops that, participants said, sold tobacco products to minors with little or no effective age-checking. One headmaster spoke of an attempt to act directly on this:

“My school was right opposite a shop that sold cool lip. From that very place, I filed a complaint myself. A team came, looked around, reported “there’s nothing here, sir,” and went away. But the shopkeeper nearby told me afterwards that before the team even arrived, he had already moved the items to his house after receiving word that the inspection is coming.” (P1).

What distinguished this theme from a simple account of weak enforcement was what participants described as happening after such a complaint. One teacher was direct when asked about the risk this poses, not to the shop but to the teacher:

“If we complain on the school side, the repercussions that come back through politicians and other channels, eventually it’s the teachers who get affected.” (P17).

A related strand of this theme concerns who introduces substances to students in the first place. One participant, explaining the usual developmental trajectory from a cross-school perspective, located the starting point not in the current student population but with older peers who have already left school:

“They look at the environment outside; older boys are involved in this, and playground peers are involved. Once two or three of them get caught up in it, it starts, and they pass it on to the next set of friends. It comes from outside. I think only their friends older by age [vayasuku meeriya nanbargal] who bring it in.” (P20).

This is not just an identification of source; but an explanation of transfer mechanism: substances flow from an external group of older peers into the school through the friendship ties of current students. This mechanism, revisited in theme 5, forms one half of the “social element” pattern, the other half being what happens when a student is excluded from school and subsequently becomes exactly the kind of older neighborhood peer described here. A parallel silence dynamic operates among students themselves:

“In cases like this, there’s another problem: the students cover for one another. They’re in a group, and even if they know who is using, they won’t say. There’s fear, or there’s friendship, and because of that, the information doesn’t reach us easily. Sometimes the younger children will tell us, the older ones are afraid of what will happen to them from their friends.” (P15).

This theme describes a perimeter that is porous in two ways at once: substances move inward from a retail environment that formal complaints find difficult to affect, and can put the complainant at risk; and information about use struggles to move inward from the peer group, for the same underlying reason; a reasonable fear of consequences.

### 5.3 Theme 3: a broad spectrum of family situations and parental responses

The third theme concerns the home environments from which students come and the range of parental responses teachers described when a concern was raised. Participants were careful to emphasize that they were describing a spectrum rather than a single type of family. At one end, participants described households shaped by the rhythm of informal-sector work, with both parents leaving before the child wakes and returning after the child sleeps, such that parents may be entirely unaware of what occurs during their child’s unsupervised hours:

“Our children are mostly lower-middle-class; about ninety percent of the parents are out working. In the morning, before the child has even left for school, the parents have already gone, and by the time the parents come back, the child is already asleep. Sometimes the parents don’t even get to see the child at all during the week. So it is only after we complain that they find out whether their child is studying or using something.” (P9).

The most common initial response to disclosure, in the middle of the spectrum, was described as protective denial, which in most cases gave way to cooperation once a teacher could present specific rather than general evidence:

“When we first tell parents, about ninety-five percent will not accept it immediately. But if we can show them clear evidence of what we found and where we found it, then slowly, many of them do accept it.” (P2).

A number of participants explicitly pushed back against any interpretation that would locate the source of the problem in ‘the family’ as a category, describing eroded supervision as a structural consequence of economic precarity that families had not created and could not easily reverse alone. This framing recurred strongly enough across the corpus to constitute a distinct interpretive position within the theme: structural empathy rather than moral attribution.

### 5.4 Theme 4: inclusive practices and the relational work of teachers

The fourth theme concerns what teachers do once a student of concern has been identified. Two registers of practice recurred across the interviews. The first is a register of structural inclusion. Teachers described actively engaging students who appeared to be drifting, drawing them into structured, valued activities like sports, the National Cadet Corps (NCC), art, dance, and gardening on the explicit reasoning that unstructured time is when usage is most likely to occur:

“If we keep them busy, the free time gets reduced and the habit comes down with it. The habit increases during free time. We get them involved in sports, NCC, dance, and yoga, we keep them occupied.” (P12).

This reasoning was independently expressed by several participants (P12, P15, P17), and reflecting a shared institutional logic, rather than an individual teaching preference. Regular attendance emerged as an important consequence of this reasoning. When asked specifically about attendance among students of concern, one teacher was unequivocal:

“They are mostly regular in attendance mostly. The reason is, he has an interest; if he comes to school, this gang forms. That is the interest he has, that is why he comes. So they’re about seventy percent regular.” (P10).

This observation, that the social ties that organize substance-use risk are also what reliably bring students to school, is the empirical core of the Attendance Paradox, which is developed in Section 6.2.

The second register concerns how teachers speak of and about identified students. Several participants independently used the term “social element” to describe what happens to a student who is removed from school: no longer a student to be guided, but a fixture in the neighborhood whom younger students may look up to, and who may himself subsequently be involved in supplying the substance he was once caught using. This framing was not presented by participants as a hypothetical risk but as an account of an outcome they believed they had repeatedly observed. It functioned as the principal rationale articulated by participants themselves for why relational inclusion was treated as a substantive institutional response rather than merely a soft alternative to discipline.

### 5.5 Theme 5: disciplinary dilemmas and what “sending him out” is understood to achieve

The fifth theme returns to formal institutional response, building on the “social element” framework from theme 4 to provide a more complete picture of how teachers and their institutions weigh the options available to them. Counseling support was described as valuable but limited, currently organized around individual crisis referrals rather than regular, preventive provision. On the transfer certificate (TC), participants described two competing pressures: internal pressure from some colleagues to use the TC quickly, and the considered resistance of headmasters who cited the “social element” outcome as the reason for restraint:

“Teachers come to me and say if you deal with this softly, this child won’t come under control; give him TC immediately and send him out immediately. But how can we do that? We are a state school. Inclusive education is education for all; every child belongs here. If I turn that child out, he becomes a social element. And then someone gets hold of him, and maybe he’s even selling these things himself. I look out for him as much as I can, and I look out for the school. I’m trying to make him a good child. If I really can’t, we have a discipline committee; all the teachers get together and talk about it. Giving TC is never a solution for me. I’d only consider it if he hurt someone or even hit a teacher; only then would I think about it and go to the officers above.” (P14).

Another participant described the same logic from the perspective of what happens after a TC is issued:

“If we give him a TC, he doesn’t stop, he just goes outside the school, often with less supervision than before. At least within the school, we can watch him and keep trying.” (P9).

“The main issue is, once he’s got the TC and gone, the boy who’s still inside, he [the one outside] gathers him in. He pulls him into his team.” (P11).

The theme concludes the empirical account by showing that participants’ reluctance to support issuing a TC was grounded in what they described as the “social element” outcome, which they reported having repeatedly witnessed following students’ removal from school.

## 6 Discussion

### 6.1 From five themes to institutional ambivalence model

#### 6.1.1 Synthesizing the five themes

The five themes collectively describe two functions that the urban government school holds simultaneously. Themes 1 and 2 describe Function A: a substance landscape that has become more concealable and easily coordinated since the COVID-19 pandemic, within a retail and social environment over which the school has limited control. Themes 3–5 describe Function B: a stable routine, a deliberate practice of recognition and inclusion, and a sustained reluctance to remove students even under pressure to do so. Collectively, the themes explain why these two functions are held together rather than one displacing the other. The recurring “social element” framing across Themes 4 and 5 represents participants’ own explanatory theory of what happens when care is withdrawn: a student removed from the school’s routine does not disappear but is repositioned from someone the school is responsible for to a fixture of the neighborhood who may subsequently draw others in.

#### 6.1.2 The institutional ambivalence model (IAM)

The Institutional Ambivalence Model (IAM; Figure 3) synthesizes the five empirically derived themes, with the theoretical frameworks presented in Section 3 providing an interpretive lens for understanding the relationships identified in the data. The model proposes that the urban government school, under the structural conditions described by Balagopalan (5, 17) and the inclusion mandate of the RTE Act, 2009, holds two functions in one institutional space: Function A (exposure to substance-use risk arising from the school’s retail, social, and digital surroundings; Theme 1-2) and Function B (a stable, caring routine provided through recognition, inclusion, and a reintegration-first stance; Theme 3–5). These functions are connected by a mediating mechanism—teachers’ relational and emotional labor (21, 22) through which both functions coexist without one displacing the other. The model’s empirical signature is the Attendance Paradox: students most exposed to Function A are, at the same time, those most consistently held within reach of Function B.

**Figure 3:**
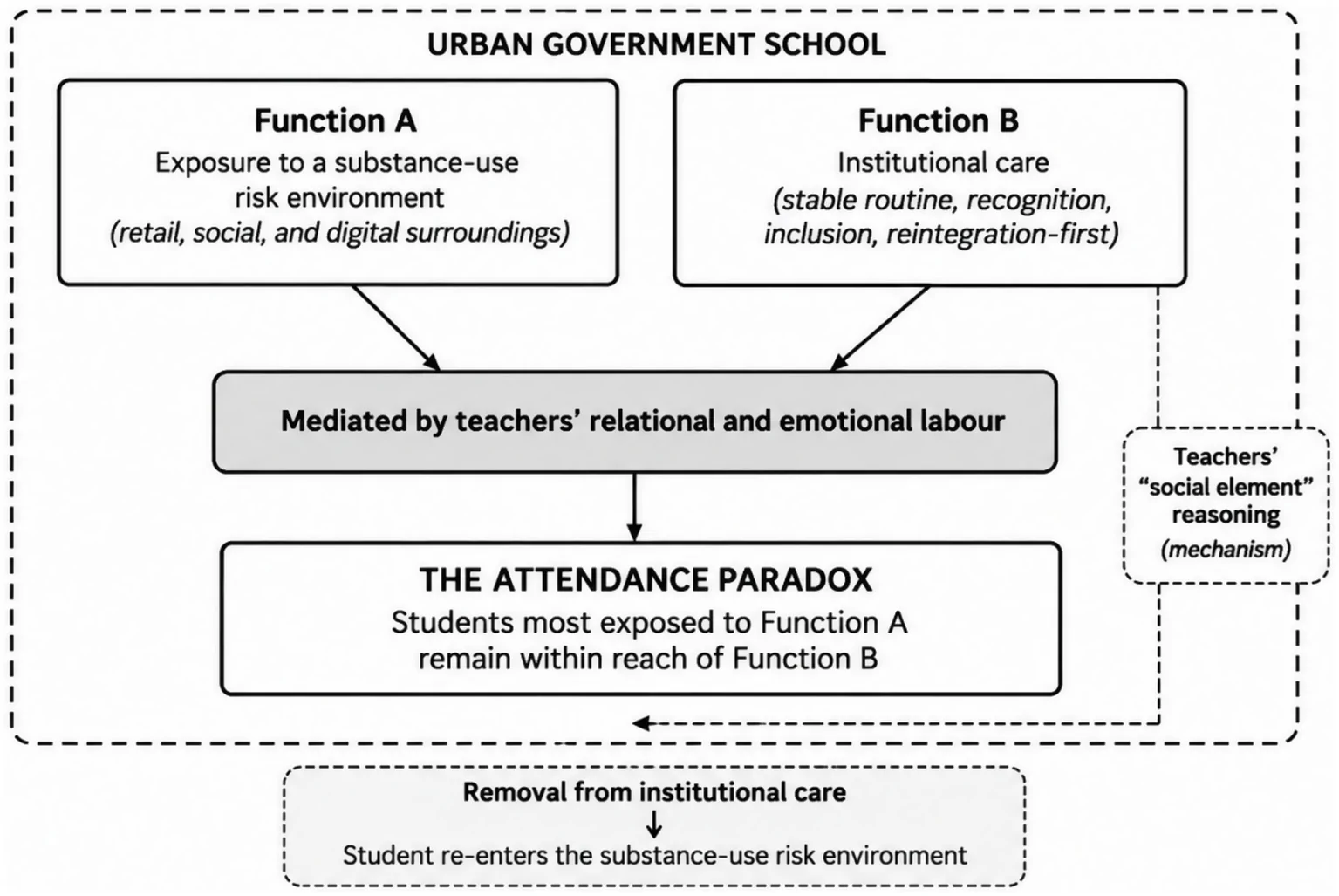
Institutional ambivalence model (IAM).

#### 6.1.3 Conceptual contribution and novelty of the IAM

The principal conceptual contribution of the present study is the development of the Institutional Ambivalence Model (IAM). The IAM was developed inductively from teachers’ accounts generated through reflexive thematic analysis. Existing theoretical frameworks were subsequently used to interpret and explain the relationships identified in the empirical data. Thus, the IAM represents an empirically grounded conceptual model that extends existing theoretical perspectives by explaining how institutional ambivalence can operate at the level of the school as an institution, rather than solely through individual social roles, within contexts of structural marginality.

This extends beyond a conceptual synthesis of existing theories in three specific ways. First, following Merton and Barber (20), it reconceptualizes sociological ambivalence by treating the simultaneous holding of two normatively conflicting functions as a structural property of an institution rather than merely of an individual role. This extends the concept of sociological ambivalence beyond the individual-role dyad to the level of institutional structure. Second, following Rhodes (18, 19), it locates Function A in the environment rather than in any student, so the model makes no claim that the school produces or is responsible for the risk to which it is exposed; instead, the school is positioned within a risk environment that it only partially controls. Third, it identifies what holds the model together: not policy or resourcing, but teachers’ sustained relational labor even under internal pressure from colleagues (Theme 5) and external conditions that make formal escalation costly (Theme 2).

#### 6.1.4 Alternative explanations

Two alternative explanations of the Attendance Paradox warrant consideration. First, the link between substance use and regular attendance may be a selection effect: students who are more socially embedded are at the same time more likely to use substances (through the same peer networks). This interpretation is not inconsistent with the IAM but would imply that peer social capital, rather than the school environment per se, is the driving mechanism. However, participants consistently pointed to the school as a place of relative safety and predictability, rather than peer groups as school-specific, suggesting that the institutional environment itself is a meaningful factor. Second, substance-using students may attend regularly simply because there are no alternative structures available to them. When home and neighborhood do not provide an organized day, school attendance becomes the default rather than a matter of choice. This interpretation is consistent with the IAM’s Function B argument and does not contradict it; in fact it strengthens the case for treating school-based retention as a resource rather than a problem.

### 6.2 The attendance paradox in context

The Attendance Paradox—that students known to use substances were described as regular attenders was contextualized in terms of the school’s relative stability compared to the home (Theme 3). Theme 1 adds a further dimension: the pandemic-era shifts (the mask as concealment, the mobile phone as coordination) did not draw students away from school. Both are described as features of what happens inside and around the school day, not as alternatives to it. For the students of greatest concern, the school is not just one safer option among many but the environment within which much of their social world, including the peer relationships through which substances circulate, is itself located. The Attendance Paradox, therefore, is not adequately captured by the phrase “school is safer than home.” It is more precisely: “the school is where this population’s social world exists, and removing a student from it does not remove him from that world, only from the part of it that includes a teacher.”

This study complements the large-scale population-level evidence from north India. Srivastava et al. (28), using data from the UDAYA cohort study in Uttar Pradesh and Bihar, find that substance use prevalence was much higher among adolescent boys who had dropped out of school than those who were currently enrolled. This is not in contradiction with the Attendance Paradox. The UDAYA findings describe adolescents who have already left formal schooling, interviewed in a household study in two north Indian states that differ in regulatory, demographic, and institutional configurations from urban Tamil Nadu. This study is about adolescents who remain enrolled as seen by their teachers in the school. These two findings describe different points in the same process: the Attendance Paradox describes the enrolled phase during which the relational care of the school sustains attendance even in the presence of substance use; the UDAYA data describe what follows when the institutional containment breaks down, the very trajectory that the “social element” mechanism in Themes 4 and 5 is concerned with preventing.

### 6.3 The porous perimeter and the regulatory paradox

Theme 2 showed that teachers perceive complaints about the retail environment as carrying personal risk, channeled informally “through politicians.” A parallel pattern operates among students: those who could disclose who is using do not, weighing up the risk of consequences against loyalty or friendship. Together, these findings describe a perimeter porous in two directions: substances moving inward from a retail environment that complaints have difficulty changing, and information moving inward from the peer group, for the same underlying reason.

This sharpens, rather than simply illustrates, Rhodes’ (18, 19) Risk Environment Framework. The policy dimension of risk here concerns not only whether enforcement occurs but whether the institution and the individuals within it can engage with enforcement without incurring informal costs. In this way, the teachers’ relational practices in Theme 4 and 5 are not only an alternative to formal disciplines but are, for some participants, the lowest-risk available channel. This dynamic is structurally produced by the regulatory paradox identified in Section 2.2: COTPA requiring the school to report, while the RTE Act requires it to retain the same students. Teachers’ hesitance to escalate formally is therefore better understood as institutional logic, a rational response to structurally contradictory obligations rather than as individual caution or indifference.

### 6.4 Implications for policy and practice

Four policy and practice implications follow directly from the empirical findings. First, consistent with Themes 3–5, the reintegration-first approach that teachers already use, structured inclusion through sports, NCC, and mentoring, should be formally recognized and resourced, not treated as an informal workaround. Prevention policy in India has concentrated on awareness campaigns delivered to students; the present findings suggest that the relational infrastructure teachers maintain is doing more preventive work than any curriculum. Second, reflecting teachers’ accounts of sustained relational engagement (Themes 3–5), preventive and counseling support should be reconfigured as regular and preventive rather than crisis-triggered, given that the school is, in many cases, the first institutional point at which a problem becomes visible to any adult at all. Third, in response to the post-pandemic digital coordination described in Theme 1, preventive strategies should address the growing role of mobile phones and digital communication in facilitating peer coordination around substance use. The near-universal access to messaging platforms acquired for online learning has created a persistent coordination infrastructure that did not exist before 2020 and that current prevention frameworks do not address. Existing school-based prevention frameworks have not sufficiently adapted to these structural changes following the COVID-19 pandemic. Finally, consistent with institutional barriers described in Theme 2, safer institutional channels are needed through which teachers can report the sale and availability of substances around school premises without bearing the personal and professional risks associated with making complaints. Without such protective reporting mechanisms, the reporting obligations under the COTPA Act are unlikely to achieve their intended public health objectives. While these recommendations are grounded in teachers’ accounts, their implications would require adequate reasoning—particularly for counseling support and protective reporting mechanisms and should be piloted before broader rollout.

### 6.5 Broader applicability of the institutional ambivalence model (IAM)

The IAM was developed from the experiences of teachers working in government schools in Chennai and is therefore grounded in a specific institutional and socio-cultural context. Nevertheless, its analytical utility may extend to educational settings that share similar structural characteristics, including schools serving socioeconomically marginalized communities, operating under strong inclusion policies, and situated within high-risk neighborhoods. Rather than proposing universal applicability, the IAM is intended as a contextually grounded conceptual model that offers a framework for understanding how schools may simultaneously function as spaces of care and exposure. Its transferability to other educational, geographical, and policy contexts should be established through future empirical research.

### 6.6 Limitations

This study has three main limitations. First, the sample was restricted to government school teachers in the Chennai district, Tamil Nadu, and the findings may not generalize to rural settings, private schools, or other Indian states with different regulatory, demographic, or institutional conditions. In addition, participants were recruited through purposive and snowball sampling; consequently, the findings may reflect the experiences of teachers who were more willing to discuss this sensitive topic. Second, the data depend entirely on teachers’ individual perceptions and subjective experiences. This is particularly true of accounts concerning political interference and the risk of institutional complaints, which could not be independently verified; these should be read as evidence of how teachers perceive and navigate their work environment, not as verified claims about any third party’s conduct. Furthermore, the study draws exclusively on teachers’ perspectives; the absence of interviews with students and parents limits the ability to compare or triangulate these accounts across different stakeholder groups. Third, the study is cross-sectional in design. Although participants described temporal sequences, students excluded from school “becoming” social elements, the design cannot establish causal or temporal ordering.

### 6.7 Future research

Future research should examine neighborhood and attendance dynamics in rural government schools and private school settings across India to test whether the patterns reported here differ from those in the urban Chennai context. Future studies should incorporate the voices of students and parents directly, to build a more complete picture of the school environment. Longitudinal designs tracking student attendance, substance use, and post-exclusion outcomes over time would help confirm whether the divergence from Western absenteeism models is sustained, and whether the “social element” mechanism described by teachers has the causal structure participants attribute to it. Future studies should also examine whether the IAM holds in private schools and in different Indian states with varying regulatory and demographic profiles.

## 7 Conclusion

This study examined how government school teachers in Chennai, India, experience, understand, and respond to adolescent substance use. Twenty-one teachers across 16 schools described a landscape that has shifted considerably in recent years: easily concealable smokeless tobacco pouches and inhalants have largely replaced older substances, while the COVID-19 pandemic introduced two enduring structural changes: masks as a new form of in-class concealment, and the mobile phones distributed for online learning as a durable coordination infrastructure among peer networks. Teachers described a school perimeter they felt they could not easily control and a reporting process that they perceived as carrying personal costs. At the same time, they described consistently going out of their way to keep at-risk students close by drawing them into sports, clubs, and small responsibilities, and resisting the pressure to issue transfer certificates because they believed, based on their repeated experiences, that removing students from school increased their vulnerability once the institutional relationship ended. Participants described such students as becoming part of the very risk landscape the school was attempting to manage.

The central theoretical contribution of this study is the Institutional Ambivalence Model (IAM), which extends the concept of sociological ambivalence from social roles to institutions (20) and proposes that the school exposed to risk is simultaneously the institution that holds these students closest. The empirical signature of this configuration is the Attendance Paradox: the students most likely to be using substances were the students who attended most regularly. Not because nothing was wrong, but because, for many of them, school was the most structured and predictable space in their lives and the only space that consistently included a caring adult. These findings suggest a policy path that recognizes and supports what teachers are already doing, prioritizing relational inclusion and reintegration rather than attempting to combat substance use through attendance tracking or exclusionary discipline.

## Data Availability

The original contributions presented in the study are included in the article/supplementary material, further inquiries can be directed to the corresponding author.
